# A comparison of random survival forest and Cox regression for prediction of mortality in patients with hemorrhagic stroke

**DOI:** 10.1186/s12911-023-02293-2

**Published:** 2023-10-13

**Authors:** Yuxin Wang, Yuhan Deng, Yinliang Tan, Meihong Zhou, Yong Jiang, Baohua Liu

**Affiliations:** 1 Department of Social Medicine and Health Education, School of Public Health, Peking University, Beijing, China; 2 Department of Neurology, Beijing Tiantan Hospital, Capital Medical University, Beijing, China; 3 China National Clinical Research Center for Neurological Diseases, Beijing, China

**Keywords:** Hemorrhagic stroke, Random survival forest, Cox regression, Intensive care unit

## Abstract

**Objective:**

To evaluate RSF and Cox models for mortality prediction of hemorrhagic stroke (HS) patients in intensive care unit (ICU).

**Methods:**

In the training set, the optimal models were selected using five-fold cross-validation and grid search method. In the test set, the bootstrap method was used to validate. The area under the curve(AUC) was used for discrimination, Brier Score (BS) was used for calibration, positive predictive value(PPV), negative predictive value(NPV), and F1 score were combined to compare.

**Results:**

A total of 2,990 HS patients were included. For predicting the 7-day mortality, the mean AUCs for RSF and Cox regression were 0.875 and 0.761, while the mean BS were 0.083 and 0.108. For predicting the 28-day mortality, the mean AUCs for RSF and Cox regression were 0.794 and 0.649, while the mean BS were 0.129 and 0.174. The mean AUCs of RSF and Cox versus conventional scores for predicting patients’ 7-day mortality were 0.875 (RSF), 0.761 (COX), 0.736 (SAPS II), 0.723 (OASIS), 0.632 (SIRS), and 0.596 (SOFA), respectively.

**Conclusions:**

RSF provided a better clinical reference than Cox. Creatine, temperature, anion gap and sodium were important variables in both models.

**Supplementary Information:**

The online version contains supplementary material available at 10.1186/s12911-023-02293-2.

## Introduction

Stroke is the leading cause of death and long-term disability worldwide [1]. 2019 global burden of disease study (GBD) data [2]shows that stroke remains to be the second leading cause of death (11.6% of deaths) and the third leading cause of disability (5.7% of total disability-adjusted life years) in the world. Hemorrhagic stroke (HS) accounts for 37.6% of all stroke types and causes 5.5 million deaths per year approximately, with about half of deaths caused by stroke due to HS. The risk of death from HS is higher compared to ischemic stroke (IS) [3], with a 30-day mortality of 13-61% [4]. In recent years, more and more stroke patients are admitted to the intensive care unit (ICU) for neurological monitoring or management of complications, and 10-30% of them are in critical condition [5]. Hence, it is of great significance to optimize the allocation of medical resources by identifying and managing high-risk groups.

Predicting the occurrence of adverse outcomes is the prerequisite for risk stratification. Risk scores are helpful tools for prediction. Many investigators have developed diverse disease risk scoring systems. Traditional scoring systems commonly used in clinical practice include acute physiology and chronic health evaluation(APACHE II) [6], sequential organ failure assessment(SOFA) [7], Oxford acute severity of illness score(OASIS) [8], and simplified acute physiology score(SAPSII) [9], which include various variables with their respective point assignment scheme [10]. However, these traditional scores are applicable to a wide population, whose effectiveness in predicting specific diseases’ prognosis is not always satisfactory [11, 12], the application of these scores in HS is limited. Many scholars have made efforts to construct predictive tools for HS. Ho and Smith et al. [13, 14] built a prediction model of HS death in the ICU by logistic regression, and stratified the risk degree of patients by calculating risk scores. However, with the increasing number of clinical examinations and diagnostic items, clinical data often present multidimensional, highly correlated, and nonlinear characteristics [15], which limits the application conditions of traditional clinical modeling methods such as logistic and Cox regression [16]. To compensate for the shortcomings of traditional analytical methods, machine learning algorithms have emerged in the era of big data [17]. Lin and Trevisi et al. [18, 19] employed common machine learning algorithms, such as support vector machine, random forest, and neural network to predict poor functional outcomes in HS patients in the hospital. Howerer, those studies only considered the probability of survival without incorporating the time dimension, by which model prediction is often imprecise [19, 20]. Random survival forest (RSF) is a derivative of the random forest algorithm in survival analysis, which can not only handle complex right-censored survival data but also analyze interactions between variables, and has been applied to pancreatic cancer [21], sepsis [22], and breast cancer [23], and its predictive performance is better than or equivalent to Cox regression in previous studies [22, 24–26], but its performance in the field of HS remains to be investigated.

Therefore, the aim of this study was to establish RSF and Cox regression models based on clinical survival data of HS patients admitted to ICU respectively, and then we evaluated the predictive effect in terms of discrimination and calibration. We also compared developed models with traditional scores with the aim of providing a reference for clinical prediction model construction and clinical decision-making.

## Materials and methods

### Data source and study participants

All data were extracted from Medical Information Mart for Intensive Care IV, v2.1 (MIMIC-IV) [27], an openly accessible critical care database, containing 256,878 patients electronic medical records, which was collected by Beth Israel Deaconess Medical Center in Boston, Massachusetts from 2008 to 2019. Patients’ demographic information, laboratory tests, vital signs, hospital status, medication and surgical procedures were documented in detail in the MIMIC-IV database. All information regarding patients’ identification were anonymized and all identifiable information were hidden. Thus, informed consent was exempt. The author had completed all the data research training from the Collaborative Institutional Training Initiative in order to obtain database permission (Record ID:52,310,626). Our study complies with the Transparent Reporting of a multivariable prediction model for Individual Prognosis Or Diagnosis (TRIPOD) guideline statement [28].

Among 69,639 ICU admission records in MIMIC-IV, 53,569 patients with first admission were selected. Included criteria were: (1) patients first diagnosed of HS(the diagnosis international classification of diseases (ICD) codes were shown in Table S1); (2) first ICU admission record. Excluded criteria were: (1) patients’ age ≤ 18; (2) length of ICU stay ≤ 24 h; (3) patients diagnosed of tumor, cancer or aids. Finally, 2,990 eligible patients were seected for the study in total.

### Data collection and outcomes

Based on previous studies [1, 3, 5, 19, 29, 30] and the characteristics of MIMIC-IV database, the extracted variables could be summarized into the following five parts: (1) demographic characteristics: insurance, marital status, admission age, weight, gender, hospital length of stay (HOSLOS), mechanical ventilation, weight; 2) comorbidities: myocardial infarction (MI), congestive heart failure (CHF), peripheral vascular disease (PVD), dementia, chronic pulmonary disease (CPD), rheumatic disease, peptic ulcer disease, mild liver disease, diabetes without chronic complication (diabetes without cc), diabetes with chronic complication (diabetes with cc), paraplegia, renal disease, severe liver disease; 3) vital signs: heart rate, diastolic blood pressure (DBP), systolic blood pressure (SBP), mean blood pressure (MBP), respiratory rate (RR), temperature, peripheral capillary oxygen saturation (Spo2); 4) laboratory examination: hematocrit, hemoglobin, platelets, white blood cell (WBC), anion gap, bicarbonate, blood urea nitrogen (BUN), calcium, chloride, creatinine, glucose, sodium, international normalized ratio (INR), Prothrombin time (PT), partial thromboplastin time (PTT), urine output; 5) conventional scoring systems: SAPSII, OASIS, SIRS, SOFA, Glasgow Coma Scale (GCS), Charlson comorbidity index (CCI). All of the above variables were recorded within 24 h after ICU admission. If repeated measurements were recorded, the mean values were used instead. For the missing values, if the missing proportion was greater than 20%, the variables will be eliminated, otherwise, the multiple imputation method would be used to fill [31].

Two primary outcomes were analysed in the study. One was the in-hospital survival status within 7 days after admission to the ICU, and the other was the in-hospital survival status within 28 days.

### Data preprocessing and statistical analysis

All data were extracted by writing structured query language (SQL) in Navicat Premium 15 software and data analysis procedures were conducted in R 4.1.2 software (R Foundation for Statistical Computing, Vienna, Austria). For continuous variables, normal distribution variables were presented as mean ± standard deviation ($\overline{\chi }\pm S$) and t-tests were used for comparisons between groups, while non-normal distribution variables were presented as median and interquartile range [*M*(*P*_25_, *P*_75_)] and Wilcoxon rank-sum test were used for comparison between groups. Categorical variables were presented as frequency and percentages and Chi-square test or Fisher’s exact test were used for comparison. A two-side *p*-value < 0.05 was considered statistically significant.

### Model construction and evaluation

#### RSF model

RSF is a non-parametric and nonlinear ensemble learning method, which is the extension of random forest method in survival data [32]. It is an adaptive process that can simulate the complex interaction between nonlinear effects and variables, and find important variables based on the variables ranking of the model’s output to reduce generalization errors [33] which makes it well adapted to complex survival data. RSF can calculate the cumulative risk function of each sample even though the assumption of proportional risk is not satisfied, and then aggregate by survival time to generate prediction results of integrated mortality [34].

The procedures to construct a RSF model are as follows: (1) Samples are randomly selected with replacement in the original data by the bootstrap method. Each sample includes 63% of the original data and 37% of the out of bag (OOB) data, and OOB is used to calculate the prediction error rate of the ensemble cumulative risk function. (2) Each bootstrapped sample grows into a survival tree. At each node of the tree, the log-rank or logrank-score splitting criteria is used to select ‘mtry’ candidate variables which make maximum survival difference between child nodes. When the number of final node events is less than ‘nodesize’, the growth of survival tree will quit. (3) The Nelson-Aalen method is used to calculate the cumulative risk for each tree, and the average value of the cumulative risks for all trees is used to estimate the total cumulative risk of the RSF model [34].

#### Cox regression model

Cox regression is a traditional survival analysis method, which can be presented as ${ln}\frac{h(t,x)}{{h}_{0}\left(t\right)}={\beta }_{1}{x}_{1}+...+{\beta }_{p}{x}_{p}$, $h(t,x)$ refers to the risk function at time ${\prime }t{\prime }$ under the influence of risk factor ${\prime }t{\prime }$, h_0_(t) is the baseline risk function at time ${\prime }t{\prime }$ when all independent variables ${\prime }{x}_{p}{\prime }$ equal to 0, which is related to time. The mortality risk of each patient is proportional, and the proportional coefficient can be expressed as: $\frac{h(t,x)}{{h}_{0}\left(t\right)}={exp}({\beta }_{1}{x}_{1}+...+{\beta }_{p}{x}_{p})$. The fitting Cox regression model is usually expressed as: $h(t,x)={h}_{0}\left(t\right){exp}({b}_{1}{x}_{1}+...+{b}_{p}{x}_{p})$.

#### Model construction

All data were randomly divided into the training set and the test set in a ratio of 7:3. In the training set, the least absolute shrinkage and selection operator (LASSO) analysis with ten-fold cross-validation was used to screen variables preliminarily, and then five-fold cross-validation was adopted. In each fold, the grid search method [35] was used to select optimal hyperparameters and the minimum depth method [36] was used to select the optimal variable subset for the construction of RSF model, while the akaike information criterion (AIC) [37] was used to choose the optimal variable subset for Cox regression. The AUC was regarded as the model evaluation index, when the AUC reached optimal value, then the final prediction model was determined.

#### Model evaluation

**Fig. 1:**
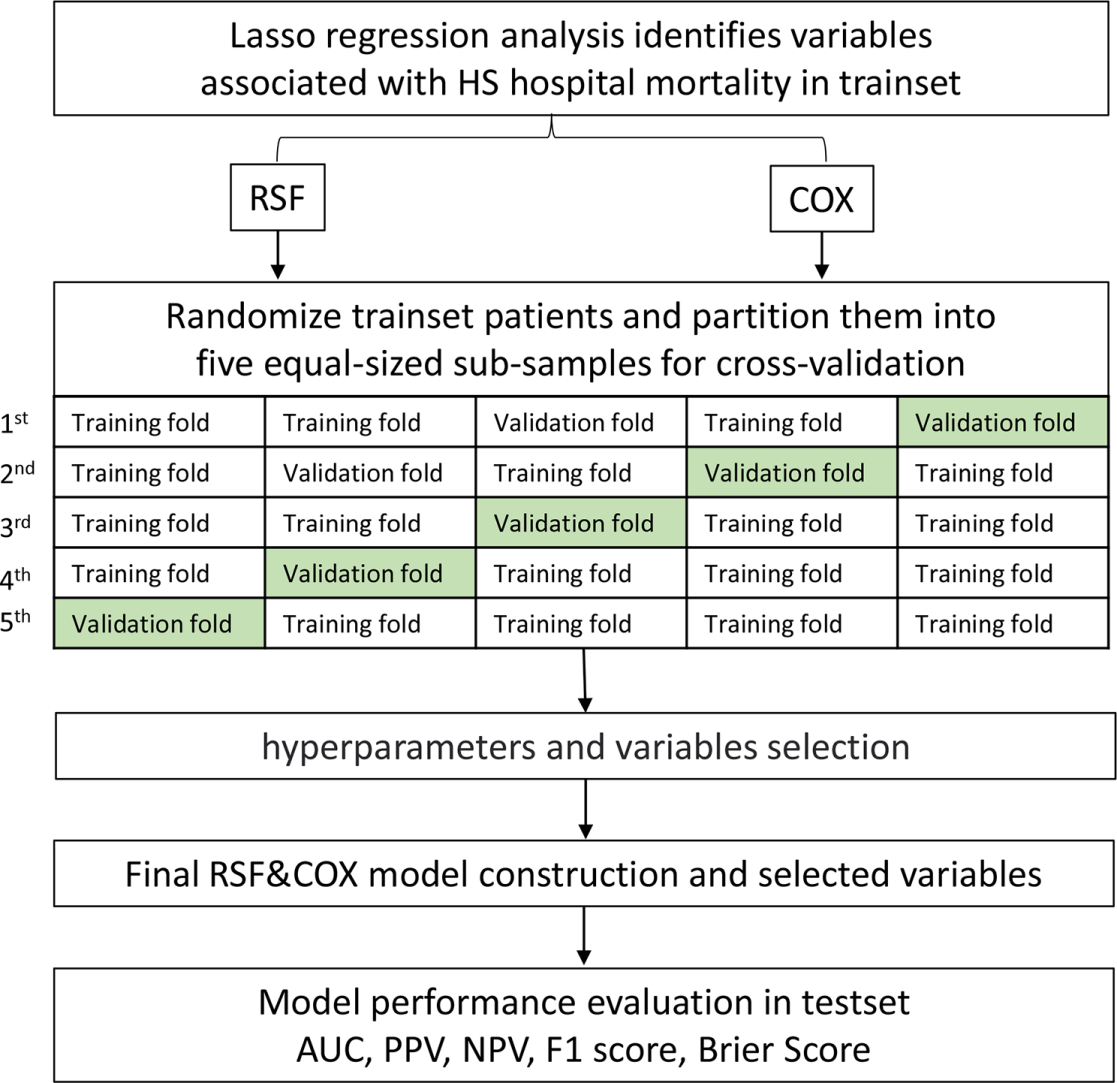
Flow chart of model construction and comparison Notes: LASSO, the least absolute shrinkage and selection operator; HS, hemorrhagic stroke; RSF, Random survival forest; AUC, area under the curve; PPV, positive predictive value; NPV, negative predictive value

In the test set, the bootstrap method [38] with 500 re-samplings was used to compare the differentiation and calibration of RSF and Cox models. The differentiation was evaluated by AUC, and the calibration was evaluated by Brier Score (BS). The larger the AUC was, the better the differentiation was, while, the smaller the BS was, the better the model fit the actual data [39]. Moreover, the positive predictive value (PPV), negative predictive value(NPV) and F1 score were also combined to compare. See Fig. 1 for the model construction and comparison process. In order to further explore the risk stratification ability of Cox and RSF model, patients were divided into low and high risk group according to the optimal cut-off point determined by Yoden index. The Kaplan-Meier (K-M) curve was drawn, and the log-rank test was used to compare whether the survival difference between the two groups was statistically significant. At the same time, the RSF and Cox models were compared with the traditional scores, namely, SAPSII, OASIS, SIRS and SOFA, which further helped to indicate that whether the constructed models had better prediction efficiency. The point assignment scheme of each system were shown in Table S2.

## Results

### Patient characteristics

**Fig. 2:**
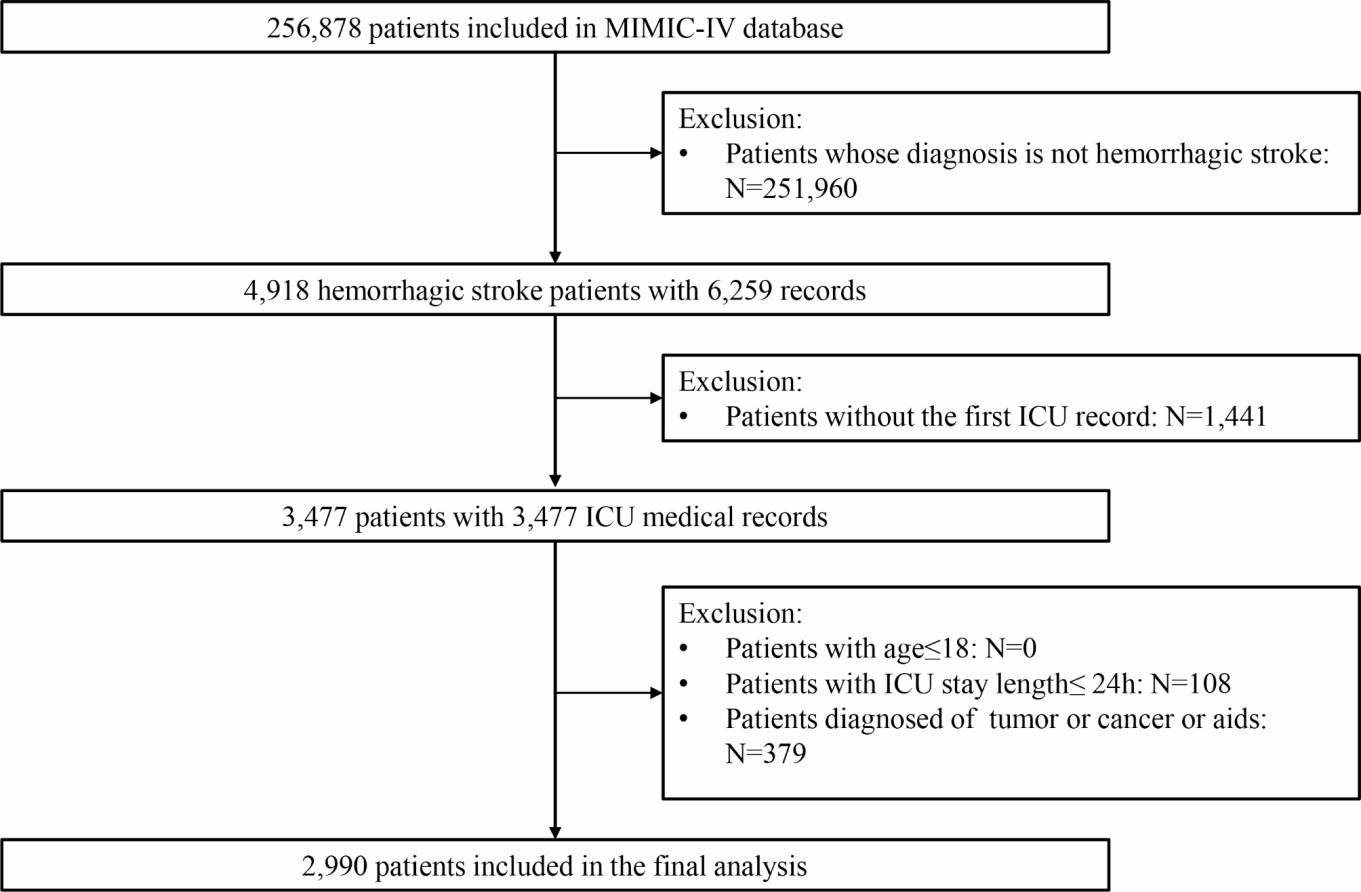
Flow chart of participants inclusion and exclusion Notes: MIMIC-IV, medical information mart for intensive care IV; ICU, intensive care unit

A total of 2,990 patients diagnosed with HS were included in our study after excluding those who did not meet the selection criteria, as shown in Fig. 2. Among these patients, 601(20.1%) deaths occurred in hospital after admission to the ICU, 376(12.6%) died within 7 days, and 586(19.6%) died within 28 days. The basic information comparison of these patients stratified by hospital-outcome was shown in Table S3. Compared with those patients who were alive, the dead patients were older(*p* < 0.001), divorced or widowed(*p* < 0.001), more likely to be diagnosed with CHF(*p* < 0.001), liver disease(*p* < 0.001), diabetes(*p* = 0.037) and renal disease(*p* < 0.001), and had a shorter length of stay in hospital. After all patients were randomly divided into the training and test set, all variables were balanced across the two data sets(*p* > 0.05), as shown in Table S2.

### Variables significance

#### LASSO regression

After LASSO analysis, a total of 19 variables were screened out from 48 variables (Figure S1): marital status, PVD, severe liver disease, diabetes without cc, admission age, heart rate, MBP, temperature, RR, platelets, WBC, anion gap, creatinine, glucose, sodium, potassium, PT, GCS, weight. The correlation analysis of the selected continuous variables showed that the correlation coefficients among all variables were lower than 0.5 (the lighter the color, the less correlated the variables were), indicating that the correlations between variables were low (Figure S2).

#### RSF model

**Fig. 3:**
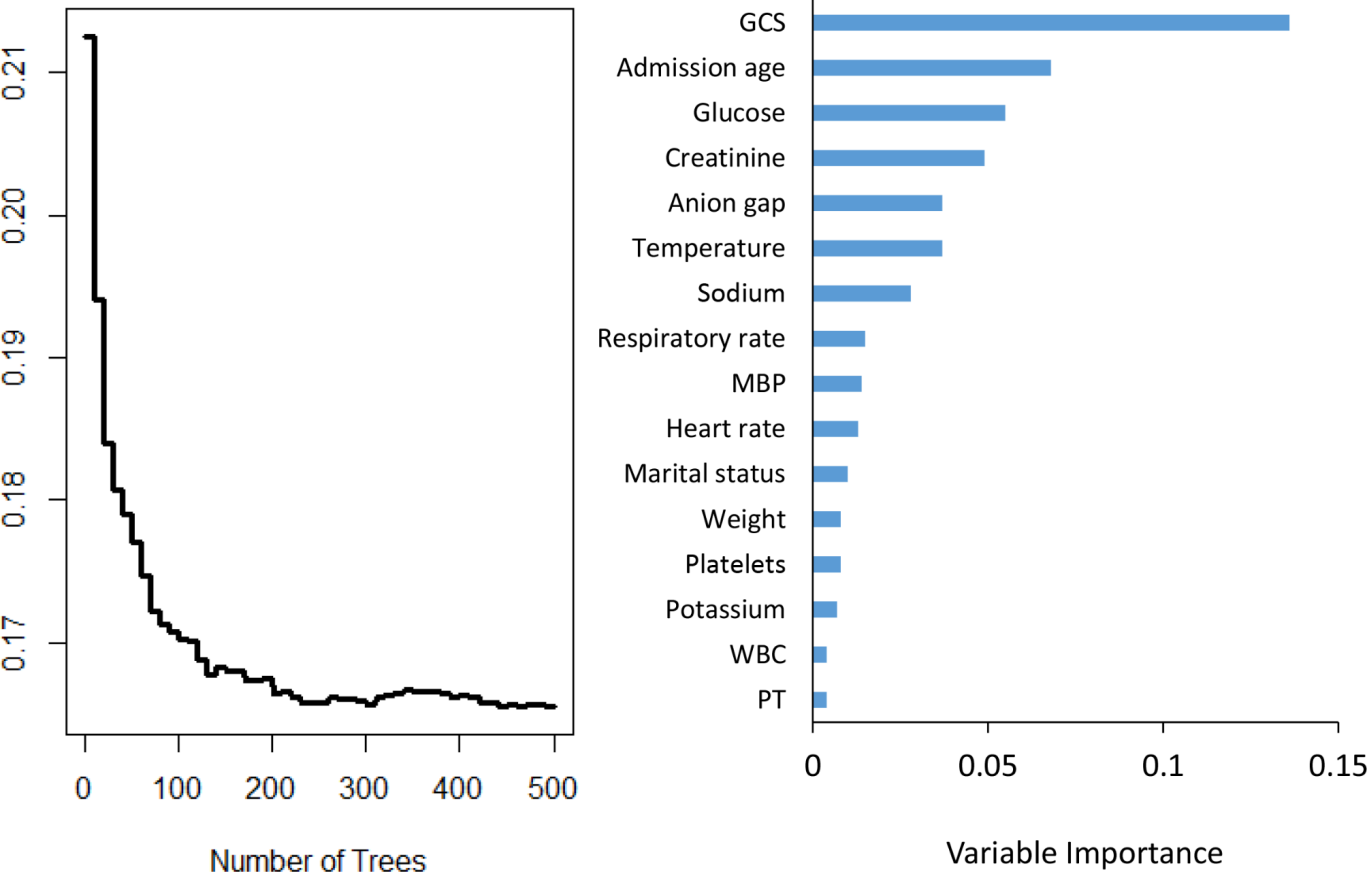
Error rate curve and variable importance of RSF Notes: MBP, mean blood pressure; WBC, white blood cell; PT, prothrombin time; GCS, glasgow coma scale

After the five-fold cross-validation and grid search in the training set, it was found that when the hyperparameter ‘ntree’ was set to be 500, ‘mtry’ was set to be 13, and ‘nodesize’ was set to be 8, the AUC of RSF model reached maximum and the error rate was low as well as stable. Finally, a total of 16 variables were selected: GCS, glucose, admission age, creatinine, temperature, anion gap, RR, sodium, MBP, marital status, heart rate, PT, platelets, potassium, weight, WBC. The error rate and variable importance were shown in Fig. 3, and the grid search process was shown in Figure S3.

#### Cox regression model

In the training set, five-fold cross-validation was adopted, and backward method was used to screen variables according to AIC. Finally, 18 variables were screened out: WBC, MBP, marital status, GCS, potassium, temperature, anion gap, PVD, severe liver disease, platelets, PT, heart rate, weight, sodium, diabetes without cc, creatinine, admission age, glucose, whose significance could be ranked by absolute value of standardized-*β*. (Table 1)

**Table 1:** Multivariate Cox regression analysis results

Characteristics	Standardized β	*HR*(95% *CI*)	*p* value
Marital status(ref:Divorced)			
Married	-0.286	0.751 [0.502, 1.123]	0.164
Single	-0.409	0.664 [0.425, 1.038]	0.073
Widowed	0.005	1.005 [0.636, 1.587]	0.984
PVD(ref:no)	-0.478	0.620 [0.418, 0.920]	0.017
Severe liver disease(ref:no)	0.832	2.299 [1.277, 4.138]	0.006
Diabetes without cc(ref:no)	-0.623	0.536 [0.389, 0.738]	< 0.001
Admission age	0.028	1.028 [1.019, 1.038]	< 0.001
Heart rate	0.014	1.014 [1.004, 1.023]	0.004
MBP	-0.010	0.990 [0.977, 1.003]	0.124
Temperature	0.239	1.270 [0.999, 1.615]	0.051
Platelets	-0.002	0.998 [0.996, 0.999]	0.011
WBC	0.022	1.022 [0.993, 1.053]	0.143
Anion gap	0.053	1.054 [1.006, 1.104]	0.027
Creatinine	0.310	1.364 [1.196, 1.554]	< 0.001
Glucose	0.010	1.010 [1.008, 1.013]	< 0.001
Sodium	0.053	1.054 [1.025, 1.085]	< 0.001
Potassium	0.249	1.282 [0.994, 1.655]	0.056
PT	0.047	1.048 [1.014, 1.083]	0.005
GCS	-0.027	0.975 [0.947, 1.003]	0.081
Weight	-0.010	0.990 [0.984, 0.997]	0.003

Notes: HR, hazard ratio; PVD, peripheral vascular disease; MBP, mean blood pressure; WBC, white blood cell; PT, prothrombin time; GCS, glasgow coma scale

### Model performance

**Fig. 4:**
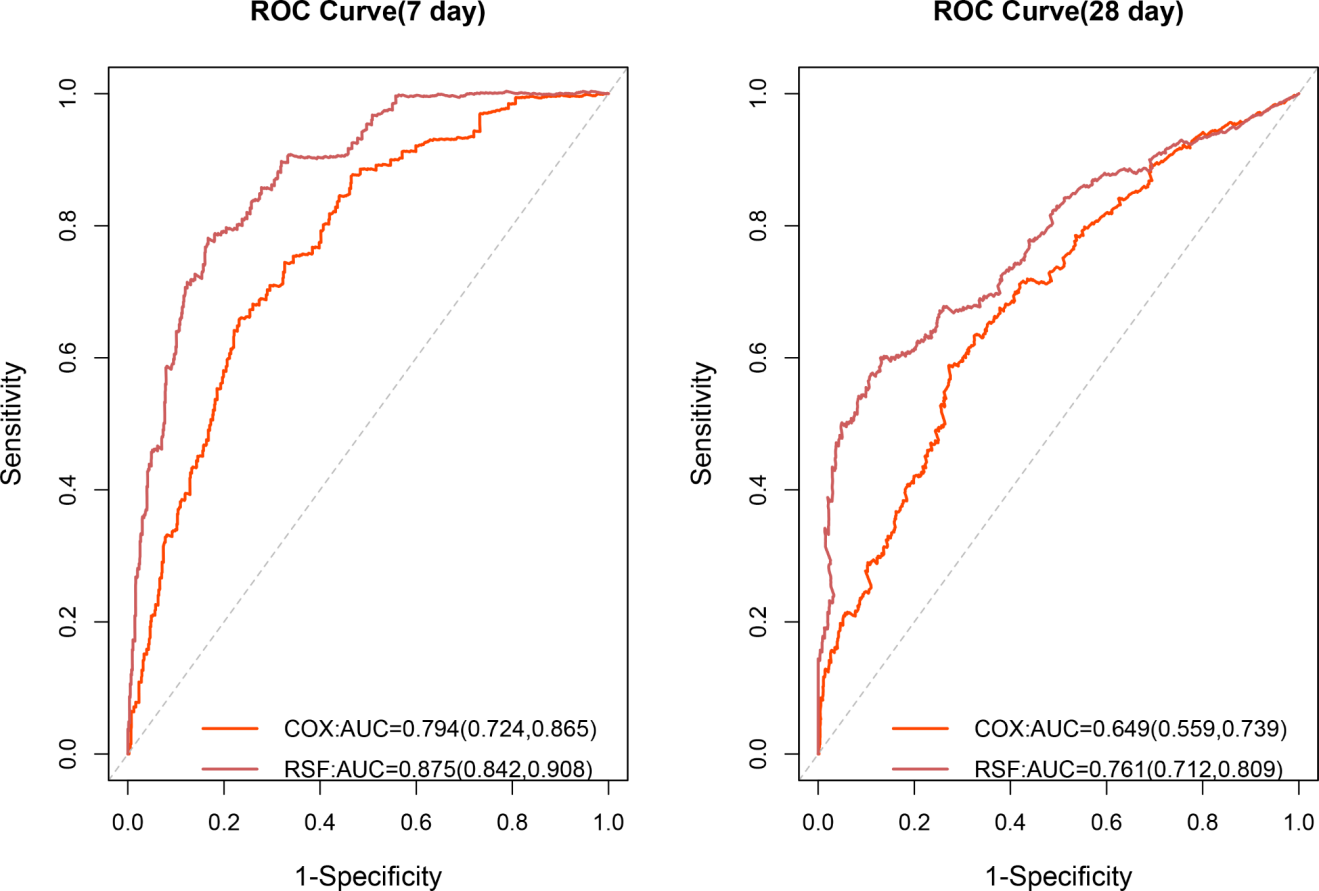
ROC curve for predicting 7-day and 28-day hospital mortality Notes: ROC, receiver operation characteristic. RSF, random survival forest

For the model differentiation, the AUCs of RSF and Cox models in predicting hospital-mortality within 7 days were 0.875(95% CI 0.842–0.908) and 0.794(95% CI 0.724–0.865) respectively, and those in predicting hospital-mortality within 28 days were 0.761(95%CI 0.712–0.809) and 0.649(95%CI 0.559–0.739) respectively. The ROC curves of RSF and Cox models in predicting 7-day and 28-day hospital mortality were shown in Fig. 4. For the model calibration, the brier scores of RSF and Cox were 0.083(95%CI 0.071–0.095) and 0.108(95%CI 0.093–0.123) in predicting hospital-mortality within 7 days, and those in predicting hospital-mortality within 28 days were 0.129(95%CI 0.116–0.143) and 0.174(95%CI 0.167–0.181) respectively. The calibration curve were shown in Figure S4. Other model performance indices, namely, PPV, NPV, F1 score, were shown in Figure S5. In addition, every performance index of the two models was statistically different(*p* < 0.001).

**Fig. 5:**
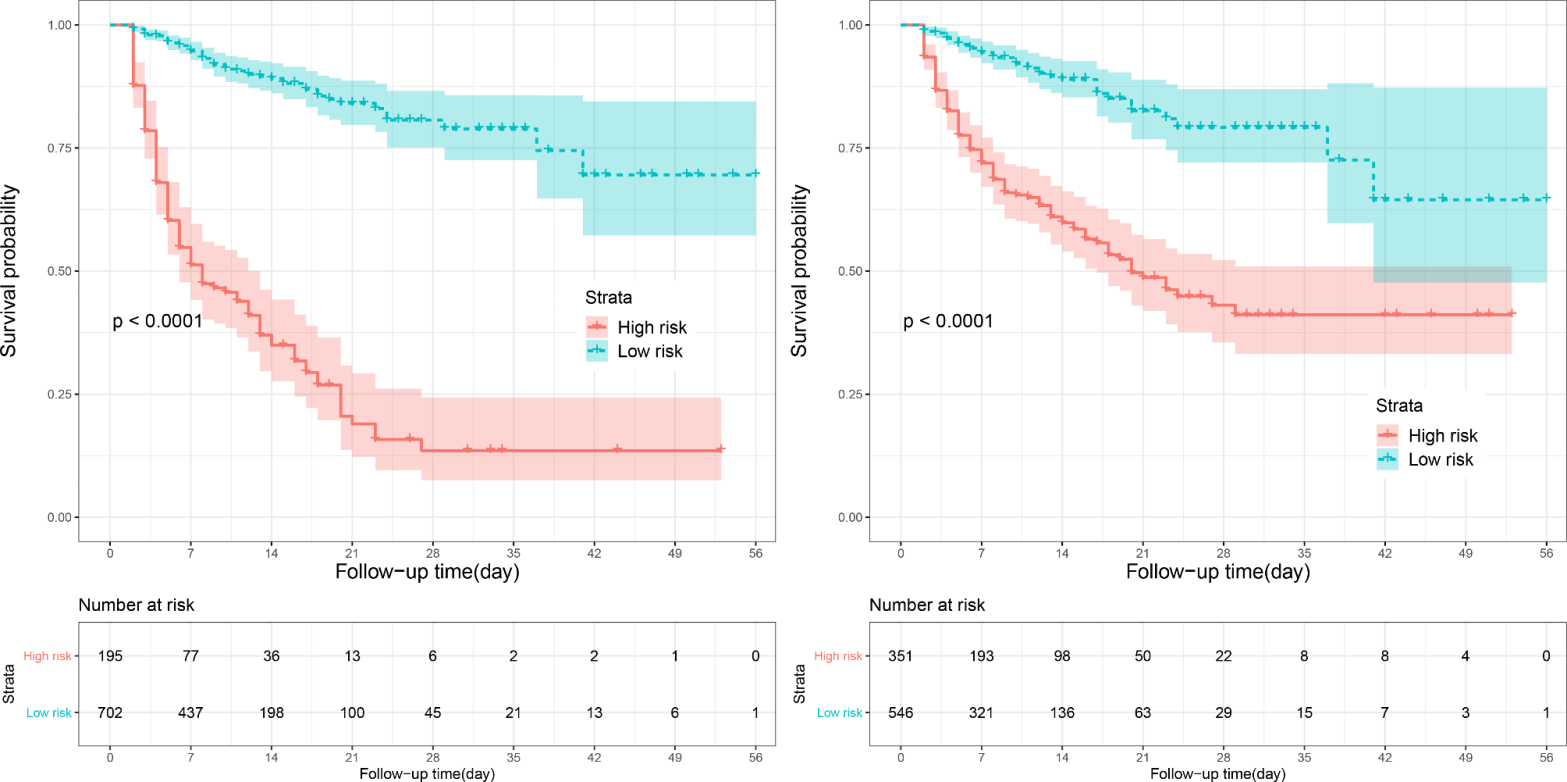
K-M curves for risk stratification. (**A**) RSF model. (**B**) Cox regression model

### Risk stratification

All HS patients were divided into high and low risk group according to optimal cut-off risk-score of RSF and Cox models. The optimal cut-off risk-score of RSF model was 22.20, while that of Cox model was 0.883, and as the patient’s risk-score increased, so did the risk of hospital death. K-M curves showed both models could divide patients into high and low risk group with significant survival difference(*p* < 0.001). (Figure 5)

### Comparison of RSF with traditional score systems

**Fig. 6:**
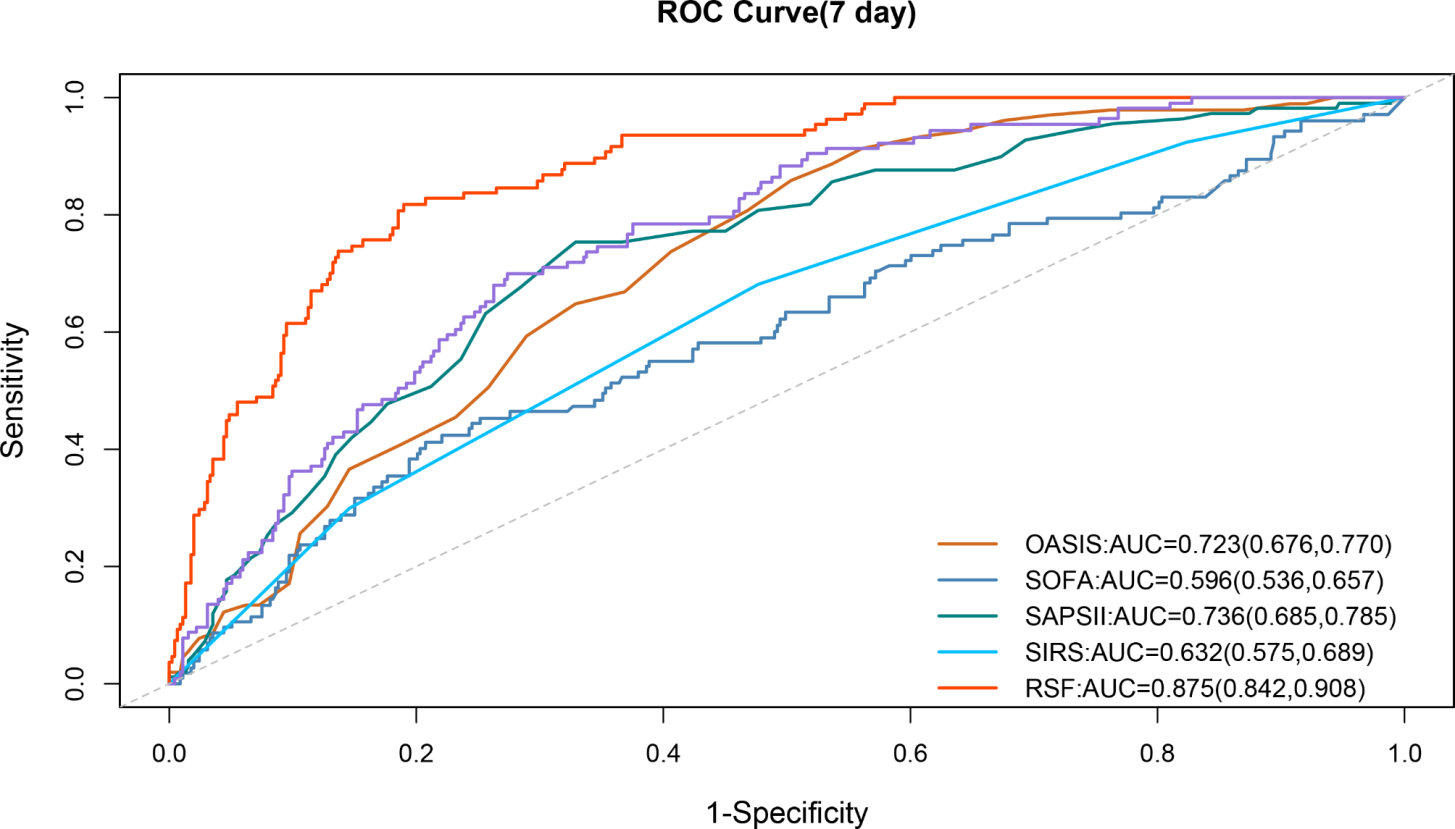
ROC curves of six models Notes: AUC, area under the curve; RSF, random survival forest; SAPSII, simplified acute physiology score; OASIS, Oxford acute severity of illness score; SIRS, systemic infammactery response syndrome score; SOFA, sequential organ failure assessment. ROC, receiver operation characteristic

As the results showed above, the 7-day prediction performance was better than that of 28-day in both RSF and Cox model. Therefore, we further compared the prediction performance of RSF and Cox models with traditional scores, namely, SAPSII, OASIS, SIRS and SOFA. After 500 re-samplings, the box scatter plot showed RSF and Cox models had a good advantage over the four traditional scores in predicting 7-day hospital mortality risk, as shown in Figure S6. The AUCs of six models were 0.875(95%CI 0.842–0.908), 0.761(95%CI 0.712–0.809), 0.736(95%CI 0.685–0.785), 0.723(95%CI 0.676–0.770), 0.632(95%CI 0.575–0.689) and 0.596(95%CI 0.536–0.657) in order from largest to smallest. Other measures related to traditional scoring systems were shown in Table S4. The ROC curves of six models for 7-day mortality were described in Fig. 6. In addition, The AUCs of six models for 28-day mortality were shown in Table S5.

## Discussion

In this study, we constructed RSF and Cox regression models to predict the risk of death of HS patients within 7 and 28 days after admission to ICU, respectively. By comparing the AUC, BS, PPV, NPV, and F1 score of the two models, it was found that RSF model was better than Cox regression model in terms of differentiation and calibration, while both models were able to divide all subjects into low-risk and high-risk groups. Comparing RSF and Cox models with traditional scores, it was observed that the AUC of RSF model was higher than that of traditional scores, suggesting that RSF had a better predictive performance and application value in disease prognosis of HS patients.

For ICU patients, studies have shown that predictive models based solely on survival status do not provide adequate information for clinicians’ intervention time decisions [40]. The dependent variables of traditional Cox regression consist of survival time and a dichotomous variable indicating whether survival outcome occurs, which takes into account the patient’s survival time but performs poorly when dealing with high-dimension clinical data because the predictive effect is easily limited by the assumption of proportional hazards [37], and can not provide clinicians with a timely decision reference in restricted time with confined medical resources. RSF can be used to generate multiple decision trees by randomizing, integrating all of them to form the final prediction model, which does not depend on applied premises such as *p*-value, proportional hazards assumption, linearity, etc., and reduces computational time by replacing cross-validation with out-of-bag data estimation. In this study, RSF had higher predictive efficacy than Cox regression for both 7-day and 28-day mortality in HS patients in the ICU. Hadanny et al. predicted 1-year mortality in patients with acute coronary syndrome, and the results also showed that compared to DeepSurv and Cox regression, the RSF model performed best (C-index = 0.924) [41], but Qiu et al. obtained the opposite conclusion (C-index = 0.611 vs. 0.629) using RSF and Cox regression respectively based on 10 characteristic variables in 82 patients with glioma [42], which may be due to the small sample size and fewer variables in that study. Lin et al. constructed RSF, DeepSurv and Cox to predict the long-term mortality of patients with ischemic stroke, the results showed that all algorithms achieved good prediction effect, which may be because the variables included were few, and these variables satisfied the application conditions of Cox regression. In this case, Cox regression is more suitable for clinical workers to apply and understand [43]. Specially, we found that RSF showed better predictive efficacy for short-term outcomes. Huang et al. also obtained similar findings when using RSF and Cox regression to predict mortality in patients with pot-belly adenocarcinoma over 1–10 years [44], which may be due to the fact that short-term mortality risk is easier to predict because it only needs to consider serious events that occur in a shorter period of time, whereas long-term mortality risk is subject to more unknown and confounding factors. In addition, compared to traditional scores, we found that the RSF model had the best AUC performance (AUC = 0.875), followed by SAPSII, OASIS, SIRS and SOFA. Zhang et al. compared RSF with SOFA, SAPS II and APS III when exploring the 30-day risk of death in sepsis patients in the ICU, and the C-index of the four scores were 0.551, 0.654, 0.669 and 0.731, all of which were lower than the RSF model [22], which was consistent with the results of our study, suggesting that the RSF may provide new methods and ideas for developing new clinical disease severity scores. Moreover, some of the scoring systems had low AUCs, and some others had the opposite in the study, which may due to the fact that SAPSII and OASIS are mainly used to assess the severity of illness and predict prognosis for the ICU or general ward patients. SIRS and SOFA are mainly used for assessing systemic inflammatory response and degree of organ dysfunction respectively.

Identifying variables that are associated with the risk of death has important implications for clinical practice. Both RSF and Cox regression were able to screen for variables of high importance, and among the top 10 important variables, the variables that were identical in both models were creatine, temperature, anon gap, and sodium. Creatine is an important indicator for monitoring of acute kidney injury [45], and Luo et al. showed a steep linear relationship between reduced blood creatine levels and increased risk of in-hospital and 1-year mortality in patients with intracranial hemorrhage when blood creatine values were < 1.9 mg/dL [46]. We found a 1.3-fold increase in the risk of in-hospital death in HS patients for each range of temperature change, which was consistent with previous studies [47]. Iglesias Rey et al. conducted a retrospective study of 887 patients with non-traumatic cerebral hemorrhage and found that patients with hypertensive cerebral hemorrhage had the highest body temperature and the greatest increase in body temperature within 24 h. Patients with hypertensive cerebral hemorrhage who developed hyperthermia after 3 months had a 5.3-fold increased risk of poor prognosis, moreover, the amount of edema within 24 h was positively correlated with body temperature in patients with cerebral hemorrhage due to hypertension [48]. Anion gap reflects the acid-base balance in body fluids and plays an important role in the identification of metabolic acidosis [49]. Previous studies have shown that anion gap is an important short- and long-term prognostic marker in patients with IS [50], however, its use in patients with HS is less studied. Shen et al. found that HS patients experienced a decrease in the mini-mental state examination, GCS and other indicators of neurological and cognitive function as the anion gap increased at the time of admission [51]. A meta-analysis had shown that high sodium intake was positively associated with stroke risk, with a 23% increase in stroke risk for every 86 mmol/d increase in sodium intake [52]. Wang et al., who included 64,909 patients with non-traumatic HS in the United States, showed that spontaneous cerebral hemorrhage patients with abnormal serum sodium had a 1.11-fold increased risk of 30-day readmission compared to patients with normal serum sodium [30].

We have some strengths in this study. We not only compared the predictive efficacy of RSF and Cox, we also compared the models constructed by RSF and Cox with the clinical traditional scoring systems, in addition, we also found the variables that had a strong influence on the occurrence of patient’s deaths in the ICU and ranked the variables in terms of importance, which may provide guidance for further practical applications. However, this study has several limitations. Firstly, the MIMIC-IV database is a single-center database, which may limit the applicability of the study results to patients in other centers, so future inclusion of clinical data from multiple centers is desired for external validation. Secondly, due to the limitations of the MIMIC-IV database features, some important indicators such as bilirubin, lactate and albumin could not be included in the analysis because of serious missing values. Finally, only demographic information, laboratory indicators, and comorbidity information were included in this study, and some important information such as medication and imaging tests were not included, which reduced the predictive performance of the models.

## Conclusion

We constructed the RSF and Cox models based on the survival data of patients with HS in the ICU. The results showed that the prediction performance of RSF was better than Cox regression for 7-day and 28-day mortality, with creatine, temperature, anion gap and sodium ranking in the top 10 important variables in both models. RSF can provide new ideas for clinical decision-making of HS patients.

### Electronic supplementary material

Below is the link to the electronic supplementary material.

Supplementary Material 1

## Data Availability

MIMIC-IV is a publicly available database. All data in this study can be found at https://physionet.org/about/database/.

## Abbreviations

AUC: Area under the curve
BUN: Blood urea nitrogen
CCI: Charlson comorbidity index
CPD: Chronic pulmonary disease
DBP: Diastolic blood pressure
GCS: Glasgow coma scale
HR: Hazard ratio
HS: Hemorrhagic stroke
INR: International normalized ratio
LASSO: Least absolute shrinkage and selection operator
LOS: Length of stay
MBP: Mean blood pressure
NPV: Negative predictive value
OASIS: Oxford acute severity of illness score
PPV: Positive predictive value
PT: Prothrombin time
PTT: Partial thromboplastin time
PVD: Peripheral vascular disease
ROC: Receiver operation characteristic
RR: Respiratory rate
RSF: Random survival forest
SAPSII: Simplified acute physiology score
SBP: Systolic blood pressure
SIRS: Systemic infammactery response syndrome score
SOFA: Sequential organ failure assessment
Spo2: Peripheral capillary oxygen saturation
WBC: White blood cell
