# Supplementary material for: A comparison of random survival forest and Cox regression for prediction of mortality in patients with hemorrhagic stroke
Source: BMC Med Inform Decis Mak. 2023 Oct 13;23:215. doi: 10.1186/s12911-023-02293-2 (PMC10576378; doi:10.1186/s12911-023-02293-2)
Supplement: Supplementary file 1 — Supplementary Material 1 [file 12911_2023_2293_MOESM1_ESM.docx]

Supplementary Material

A comparison of random survival forest and Cox regression for prediction of mortality in patients with hemorrhagic stroke

Yuxin Wang^1^, Yuhan Deng^1^, Yinliang Tan^1^, Meihong Zhou^1^, Yong Jiang^2, 3*^,Baohua Liu^1, *^

^1^Department of Social Medicine and Health Education, School of Public Health, Peking University, Beijing, China

^2^Department of Neurology, Beijing Tiantan Hospital, Capital Medical University, Beijing, China

^3^China National Clinical Research Center for Neurological Diseases, Beijing, China

*** Correspondence:**Baohua Liu, Yong Jiang
[baohualiu@bjmu.edu.cn](mailto:baohualiu@bjmu.edu.cn), jy78@vip.sina.com

Table S1. Diagnostic information of patients with hemorrhagic stroke.

| ICD code | ICD version | Long title |
| --- | --- | --- |
| 430 | 9 | Subarachnoid hemorrhage |
| 431 | 9 | Intracerebral hemorrhage |
| 4320 | 9 | Nontraumatic extradural hemorrhage |
| 4321 | 9 | Subdural hemorrhage |
| 4329 | 9 | Unspecified intracranial hemorrhage |
| I60 | 10 | Nontraumatic subarachnoid hemorrhage |
| I600 | 10 | Nontraumatic subarachnoid hemorrhage from carotid siphon and bifurcation |
| I6000 | 10 | Nontraumatic subarachnoid hemorrhage from unspecified carotid siphon and bifurcation |
| I6001 | 10 | Nontraumatic subarachnoid hemorrhage from right carotid siphon and bifurcation |
| I6002 | 10 | Nontraumatic subarachnoid hemorrhage from left carotid siphon and bifurcation |
| I601 | 10 | Nontraumatic subarachnoid hemorrhage from middle cerebral artery |
| I6010 | 10 | Nontraumatic subarachnoid hemorrhage from unspecified middle cerebral artery |
| I6011 | 10 | Nontraumatic subarachnoid hemorrhage from right middle cerebral artery |
| I6012 | 10 | Nontraumatic subarachnoid hemorrhage from left middle cerebral artery |
| I602 | 10 | Nontraumatic subarachnoid hemorrhage from anterior communicating artery |
| I6020 | 10 | Nontraumatic subarachnoid hemorrhage from unspecified anterior communicating artery |
| I6021 | 10 | Nontraumatic subarachnoid hemorrhage from right anterior communicating artery |
| I6022 | 10 | Nontraumatic subarachnoid hemorrhage from left anterior communicating artery |
| I603 | 10 | Nontraumatic subarachnoid hemorrhage from posterior communicating artery |
| I6030 | 10 | Nontraumatic subarachnoid hemorrhage from unspecified posterior communicating artery |
| I6031 | 10 | Nontraumatic subarachnoid hemorrhage from right posterior communicating artery |
| I6032 | 10 | Nontraumatic subarachnoid hemorrhage from left posterior communicating artery |
| I604 | 10 | Nontraumatic subarachnoid hemorrhage from basilar artery |
| I605 | 10 | Nontraumatic subarachnoid hemorrhage from vertebral artery |
| I6050 | 10 | Nontraumatic subarachnoid hemorrhage from unspecified vertebral artery |
| I6051 | 10 | Nontraumatic subarachnoid hemorrhage from right vertebral artery |
| I6052 | 10 | Nontraumatic subarachnoid hemorrhage from left vertebral artery |
| I606 | 10 | Nontraumatic subarachnoid hemorrhage from other intracranial arteries |
| I607 | 10 | Nontraumatic subarachnoid hemorrhage from unspecified intracranial artery |
| I608 | 10 | Other nontraumatic subarachnoid hemorrhage |
| I609 | 10 | Nontraumatic subarachnoid hemorrhage, unspecified |
| I61 | 10 | Nontraumatic intracerebral hemorrhage |
| I610 | 10 | Nontraumatic intracerebral hemorrhage in hemisphere, subcortical |
| I611 | 10 | Nontraumatic intracerebral hemorrhage in hemisphere, cortical |
| I612 | 10 | Nontraumatic intracerebral hemorrhage in hemisphere, unspecified |
| I613 | 10 | Nontraumatic intracerebral hemorrhage in brain stem |
| I614 | 10 | Nontraumatic intracerebral hemorrhage in cerebellum |
| I615 | 10 | Nontraumatic intracerebral hemorrhage, intraventricular |
| I616 | 10 | Nontraumatic intracerebral hemorrhage, multiple localized |
| I618 | 10 | Other nontraumatic intracerebral hemorrhage |
| I619 | 10 | Nontraumatic intracerebral hemorrhage, unspecified |
| I62 | 10 | Other and unspecified nontraumatic intracranial hemorrhage |
| I620 | 10 | Nontraumatic subdural hemorrhage |
| I6200 | 10 | Nontraumatic subdural hemorrhage, unspecified |
| I6201 | 10 | Nontraumatic acute subdural hemorrhage |
| I6202 | 10 | Nontraumatic subacute subdural hemorrhage |
| I6203 | 10 | Nontraumatic chronic subdural hemorrhage |
| I621 | 10 | Nontraumatic extradural hemorrhage |
| I629 | 10 | Nontraumatic intracranial hemorrhage, unspecified |
| 430 | 9 | Subarachnoid hemorrhage |
| 431 | 9 | Intracerebral hemorrhage |
| 4320 | 9 | Nontraumatic extradural hemorrhage |
| 4321 | 9 | Subdural hemorrhage |
| 4329 | 9 | Unspecified intracranial hemorrhage |
| I60 | 10 | Nontraumatic subarachnoid hemorrhage |
| I600 | 10 | Nontraumatic subarachnoid hemorrhage from carotid siphon and bifurcation |
| I6000 | 10 | Nontraumatic subarachnoid hemorrhage from unspecified carotid siphon and bifurcation |
| I6001 | 10 | Nontraumatic subarachnoid hemorrhage from right carotid siphon and bifurcation |
| I6002 | 10 | Nontraumatic subarachnoid hemorrhage from left carotid siphon and bifurcation |
| I601 | 10 | Nontraumatic subarachnoid hemorrhage from middle cerebral artery |
| I6010 | 10 | Nontraumatic subarachnoid hemorrhage from unspecified middle cerebral artery |
| I6011 | 10 | Nontraumatic subarachnoid hemorrhage from right middle cerebral artery |
| I6012 | 10 | Nontraumatic subarachnoid hemorrhage from left middle cerebral artery |
| I602 | 10 | Nontraumatic subarachnoid hemorrhage from anterior communicating artery |

Notes: ICD, international classification of diseases.

**Table S2** The point assignment scheme of each scoring system

| **Models** | **Variables** | **Scores** |
| --- | --- | --- |
| SAPSⅡ | Age | <40→0  40-59→7  60-69→12  70-74→15  75-79→16  >=90→18 |
|  | Heart Rate(HR) | 70-119→0  40-69→2  120-169→4  >=160→7  <40→11 |
|  | Systolic blood pressure(SBP) | 100-199→0  >=200→2  70-99→5  <70→13 |
|  | Temperature | <39→0  >=39→3 |
|  | PaO2/FiO2 | >=200→6  100-199→9  <100→11 |
|  | Uine volume | >=1.0→0  0.05-0.999→4  <0.5→11 |
|  | Bood urea nitrogen | <10.5→0  10.5-31.0→4  >=32.0→11 |
|  | White blood cell count | 1.0-19.9→0  >=20.0→3  <1.0→12 |
|  | Potassium | 3.0-4.9→0  <3 or >=5→3 |
|  | Sodium | 125-144→0  >=145→1  <125→5 |
|  | Bicarbonate | >=20→0  15-19→3  <15→6 |
|  | Bilirubin | <68.4→0  68.4-102.5→4  >=102.6→9 |
|  | GCS | 14-15→0  11-13→5  9-10→7  6-8→13 |
|  | Chronic disease | metastatic cancer→9  hematological malignancy→10  Aids→17 |
|  | ICU type | selective operation→0  medical patients→6  emergency operation→8 |
| OASIS | Pre- ICU length of stay(pre-ICU LOS) | <0.17→5  0.17-4.94→3  4.95-24.00→0  24.01-311.80→2  >311.80→1 |
|  | Age | <24→0  24-53→3  54-77→6  78-89→9  >90→7 |
|  | GCS | 3-7→10  8-13→4  14→3  15→0 |
|  | Heart rate(HR) | <33→4  33-88→0  89-106→1  107-125→3  >125→6 |
|  | Mean arterial pressure(MAP) | <20.65→4  20.65-50.99→3  51.00-61.32→2  61.33-143.44→0  >143.44→3 |
|  | Respiratory rate(RR) | <6→10  6-12→1  13-22→0  23-30→1  31-44→6  >44→9 |
|  | Temperature | <33.22→3  33.22-35.93→4  35.94-36.39→2  36.40-36.88→0  36.89-39.88→2  >39.88→6 |
|  | Urine output | <671→10  671-1426.99→5  1427.00-2543.99→1  2544.00-6896→0  >6896→8 |
|  | Ventilated | NO→0  YES→9 |
|  | Elective surgery | NO→6  YES→0 |
| SIRS | Temperature | Temperature_min<36.0 or Temperature_max>38.0→1  Else→0 |
|  | Heart rate | Heart rate_max>90.0→1  Else→0 |
|  | Respiratory rate | Respiratory rate_max>20.0→1  Else→0 |
|  | Paco2 | Paco2_min<32.0→1  Else→0 |
|  | White blood cell count(WBC) | WBC_min<4.0 or WBC_max>12.0 →1  Else→0 |
|  | Immature neutrophils | Immature neutrophils>10%→1  Else→0 |
| SOFA | PaO2/FiO2 | >=400→0  <400→1  <300→2  <200→3  <100→4 |
|  | Platelets | >=150→0  <150→1  <100→2  <50→3  <20→4 |
|  | Bilirubin | <1.2→0  1.2-1.9→1  2.0-5.9→2  6.0-11.9→3  >12.0→4 |
|  | Cardiovascular function | Mean arterial pressure>=70mmHg→0  Mean arterial pressure<=70mmHg→1  Dopamine<=5 or dobutamine(any dose)→2  5<Dopamine<15 or epinephrine<=0.1 or norepinephrine<=0.1→3  Dopamine>15 or epinephrine>0.1 or norepinephrine>0.1→4 |
|  | GCS | 15→0  13-14→1  10-12→2  6-9→3  <6→4 |
|  | Creatinine | <1.2→0  1.2-1.9→1  2.0-3.4→2  3.5-4.9→3  >5.0→4 |
|  | Urine output | <500→3  <200→4 |

**Table S**3 Baseline characteristics of the included patients.

| Characteristics | Overall  (n=2990) | Alive  (n=2389) | Dead  (n=601) | *p* value | test-set  (n=897) | train-set  (n=2093) | *p* value |
| --- | --- | --- | --- | --- | --- | --- | --- |
| Insurance, n(%) |  |  |  | <0.001 |  |  | 0.655 |
| Medicaid | 167 (5.6) | 141 (5.9) | 26 (4.3) |  | 49 (5.5) | 118 (5.6) |  |
| Medicare | 1258 (42.1) | 957 (40.1) | 301 (50.1) |  | 367 (40.9) | 891 (42.6) |  |
| Other | 1565 (52.3) | 1291 (54.0) | 274 (45.6) |  | 481 (53.6) | 1084 (51.8) |  |
| Marital status, n(%) |  |  |  | <0.001 |  |  | 0.739 |
| Divorced | 237 (7.9) | 184 (7.7) | 53 (8.8) |  | 77 (8.6) | 160 (7.6) |  |
| Married | 1593 (53.3) | 1289 (54.0) | 304 (50.6) |  | 471 (52.5) | 1122 (53.6) |  |
| Single | 734 (24.5) | 612 (25.6) | 122 (20.3) |  | 216 (24.1) | 518 (24.7) |  |
| Widowed | 426 (14.2) | 304 (12.7) | 122 (20.3) |  | 133 (14.8) | 293 (14.0) |  |
| Admission age, years | 67.44 (16.04) | 66.21 (16.16) | 72.35 (14.54) | <0.001 | 66.95 (15.79) | 67.65 (16.14) | 0.272 |
| Gender = M, n(%) | 1560 (52.2) | 1253 (52.4) | 307 (51.1) | 0.580 | 455 (50.7) | 1105 (52.8) | 0.318 |
| Weight, Kg | 76.00 (64.06-89.70) | 76.30 (64.70-90.00) | 74.77 (61.60-87.00) | 0.006 | 76.00 (64.40-89.70) | 76.05 (64.00-89.70) | 0.738 |
| Mechanical ventilation, n(%) | 1965 (65.7) | 1463 (61.2) | 502 (83.5) | <0.001 | 588 (65.6) | 1377 (65.8) | 0.933 |
| Status = Dead, n(%) | 601 (20.1) | - | - | - | 180 (20.1) | 421 (20.1) | 1.000 |
| Status_7d=Dead, n(%) | 376 (12.6) | - | - | - | 114 (12.7) | 262 (12.5) | 0.933 |
| Status_28d=Dead, n(%) | 586 (19.6) | - | - | - | 177 (19.7) | 409 (19.5) | 0.944 |
| HOSLOS, days | 8.00 (4.00-14.00) | 8.00 (5.00-15.00) | 6.00 (3.00-11.00) | <0.001 | 8.00 (4.00-14.00) | 8.00 (4.00-14.00) | 0.370 |
| Heart rate, bmp | 77.81 (69.65-87.25) | 76.63 (69.04-85.74) | 82.52 (73.25-93.03) | <0.001 | 77.89 (69.52-87.15) | 77.77 (69.65-87.36) | 0.919 |
| SBP, mmHg | 129.82 (120.64-138.13) | 129.71 (120.88-138.21) | 130.22 (118.84-137.87) | 0.512 | 130.11 (119.96-138.23) | 129.67 (120.84-138.00) | 0.985 |
| DBP, mmHg | 66.06 (59.21-73.54) | 66.65 (60.00-74.22) | 63.33 (56.28-70.60) | <0.001 | 65.67 (59.03-73.58) | 66.14 (59.29-73.48) | 0.763 |
| MBP, mmHg | 83.88 (77.37-90.37) | 84.50 (77.90-90.70) | 81.76 (75.33-88.38) | <0.001 | 83.86 (77.14-90.60) | 83.90 (77.49-90.26) | 0.916 |
| RR, times/min | 18.00 (16.29-20.00) | 17.85 (16.17-19.73) | 18.83 (17.00-21.23) | <0.001 | 18.10 (16.30-20.19) | 17.96 (16.28-19.92) | 0.452 |
| Temperature, ℃ | 36.94 (36.72-37.24) | 36.92 (36.72-37.19) | 37.08 (36.70-37.46) | <0.001 | 36.94 (36.72-37.23) | 36.93 (36.72-37.24) | 0.898 |
| Spo2, % | 97.32 (96.00-98.63) | 97.15 (95.92-98.44) | 98.07 (96.42-99.23) | <0.001 | 97.40 (96.03-98.60) | 97.29 (95.97-98.64) | 0.835 |
| Hematocrit, % | 37.14 (33.75-40.52) | 37.35 (33.95-40.65) | 36.40 (32.58-40.10) | 0.001 | 37.10 (33.33-40.70) | 37.20 (33.90-40.47) | 0.677 |
| Hemoglobin, g/dL | 12.40 (11.20-13.60) | 12.45 (11.35-13.62) | 12.10 (10.87-13.30) | <0.001 | 12.40 (11.00-13.60) | 12.40 (11.30-13.55) | 0.674 |
| Platelets, 10^9^/L | 211.00 (170.00-259.00) | 214.67 (174.67-260.67) | 197.00 (151.00-246.67) | <0.001 | 207.00 (168.67-256.00) | 212.33 (170.50-259.67) | 0.191 |
| WBC, 10^9^/L | 10.28 (8.07-13.15) | 9.90 (7.90-12.60) | 12.00 (9.25-15.15) | <0.001 | 10.40 (8.17-13.47) | 10.20 (8.05-13.05) | 0.368 |
| Anion gap, mEq/L | 14.67 (13.00-16.50) | 14.50 (13.00-16.33) | 15.50 (14.00-17.50) | <0.001 | 14.67 (13.00-16.50) | 14.67 (13.00-16.67) | 0.684 |
| Bicarbonate, mEq/L | 23.50 (21.67-25.50) | 23.75 (22.00-25.50) | 23.00 (21.00-25.00) | <0.001 | 23.50 (21.50-25.50) | 23.50 (21.75-25.50) | 0.945 |
| BUN, mg/dL | 15.67 (12.00-21.00) | 15.00 (11.50-20.00) | 18.50 (14.00-27.00) | <0.001 | 16.00 (11.67-21.00) | 15.67 (12.00-21.00) | 0.939 |
| Calcium, mg/dL | 8.70 (8.35-9.10) | 8.75 (8.40-9.10) | 8.60 (8.20-9.05) | <0.001 | 8.70 (8.35-9.10) | 8.75 (8.35-9.10) | 0.371 |
| Chloride, mmol/L | 104.00 (101.50-106.67) | 104.00 (101.50-106.50) | 104.50 (101.50-108.00) | 0.004 | 104.00 (102.00-106.75) | 104.00 (101.50-106.50) | 0.270 |
| Creatinine, mg/dL | 0.87 (0.70-1.10) | 0.85 (0.70-1.05) | 0.95 (0.75-1.30) | <0.001 | 0.87 (0.70-1.10) | 0.87 (0.70-1.10) | 0.972 |
| Glucose, mg/dL | 130.00 (110.00-157.00) | 125.50 (107.00-148.50) | 153.00 (129.00-187.00) | <0.001 | 130.00 (109.50-159.25) | 130.00 (110.00-156.00) | 0.345 |
| Sodium, mmol/L | 139.67 (137.52-142.00) | 139.50 (137.50-141.50) | 140.00 (138.00-143.17) | <0.001 | 139.50 (137.67-142.00) | 139.75 (137.50-142.00) | 0.664 |
| Potassium, mEq/L | 3.95 (3.70-4.25) | 3.95 (3.70-4.20) | 4.00 (3.70-4.33) | 0.007 | 3.95 (3.66-4.25) | 3.97 (3.70-4.25) | 0.312 |
| INR | 1.10 (1.05-1.25) | 1.10 (1.05-1.20) | 1.15 (1.07-1.33) | <0.001 | 1.10 (1.05-1.27) | 1.10 (1.05-1.25) | 0.363 |
| PT, second | 12.50 (11.50-13.90) | 12.40 (11.50-13.67) | 12.90 (11.80-14.65) | <0.001 | 12.50 (11.50-14.10) | 12.50 (11.50-13.80) | 0.576 |
| PTT, second | 28.05 (25.60-31.17) | 28.05 (25.60-31.00) | 28.05 (25.43-32.00) | 0.465 | 28.10 (25.70-31.27) | 28.00 (25.55-31.10) | 0.221 |
| PVD, n(%) | 239 (8.0) | 196 (8.2) | 43 (7.2) | 0.445 | 71 (7.9) | 168 (8.0) | 0.977 |
| Dementia, n(%) | 166 (5.6) | 128 (5.4) | 38 (6.3) | 0.410 | 46 (5.1) | 120 (5.7) | 0.565 |
| CPD, n(%) | 404 (13.5) | 325 (13.6) | 79 (13.1) | 0.820 | 134 (14.9) | 270 (12.9) | 0.151 |
| Rheumatic disease, n(%) | 70 (2.3) | 60 (2.5) | 10 (1.7) | 0.281 | 20 (2.2) | 50 (2.4) | 0.895 |
| Peptic ulcer disease, n(%) | 19 (0.6) | 15 (0.6) | 4 (0.7) | 1.000 | 5 (0.6) | 14 (0.7) | 0.920 |
| Mild liver disease, n(%) | 133 (4.4) | 90 (3.8) | 43 (7.2) | <0.001 | 39 (4.3) | 94 (4.5) | 0.938 |
| Diabetes without cc, n(%) | 551 (18.4) | 422 (17.7) | 129 (21.5) | 0.037 | 180 (20.1) | 371 (17.7) | 0.144 |
| Diabetes with cc, n(%) | 165 (5.5) | 121 (5.1) | 44 (7.3) | 0.039 | 55 (6.1) | 110 (5.3) | 0.382 |
| Paraplegia, n(%) | 897 (30.0) | 706 (29.6) | 191 (31.8) | 0.310 | 259 (28.9) | 638 (30.5) | 0.403 |
| Renal disease, n(%) | 343 (11.5) | 244 (10.2) | 99 (16.5) | <0.001 | 107 (11.9) | 236 (11.3) | 0.652 |
| Severe liver disease, n(%) | 46 (1.5) | 25 (1.0) | 21 (3.5) | <0.001 | 15 (1.7) | 31 (1.5) | 0.820 |
| CCI | 5.00 (4.00-7.00) | 5.00 (4.00-7.00) | 6.00 (5.00-8.00) | <0.001 | 5.00 (4.00-7.00) | 5.00 (4.00-7.00) | 0.895 |
| GCS | 13.00 (8.00-14.00) | 13.00 (9.00-14.00) | 8.00 (4.00-15.00) | <0.001 | 13.00 (8.00-14.00) | 13.00 (8.00-14.00) | 0.291 |
| SAPSⅡ | 31.00 (24.00-39.00) | 29.00 (23.00-36.00) | 40.00 (33.00-49.00) | <0.001 | 31.00 (24.00-38.00) | 31.00 (24.00-39.00) | 0.111 |
| OASIS | 32.00 (26.00-39.00) | 30.00 (24.00-36.00) | 40.00 (34.00-45.00) | <0.001 | 32.00 (25.00-38.00) | 32.00 (26.00-39.00) | 0.068 |
| SIRS | 2.00 (2.00-3.00) | 2.00 (1.00-3.00) | 3.00 (2.00-4.00) | <0.001 | 2.00 (2.00-3.00) | 2.00 (2.00-3.00) | 0.984 |
| SOFA | 2.06 (1.17-3.25) | 1.86 (1.02-2.88) | 3.43 (2.04-4.80) | <0.001 | 2.08 (1.23-3.15) | 2.04 (1.15-3.29) | 0.943 |

Notes: HOSLOS, hospital length of stay; SBP, systolic blood pressure; DBP, diastolic blood pressure; MBP, mean blood pressure; RR, respiratory rate; Spo2, peripheral capillary oxygen saturation; WBC, white blood cell; BUN, blood urea nitrogen; INR, international normalized ratio; PT, prothrombin time; PTT, partial thromboplastin time; PVD, peripheral vascular disease; CPD, chronic pulmonary disease; CCI, charlson comorbidity index; GCS, glasgow coma scale; SAPSⅡ, simplified acute physiology score; OASIS, Oxford acute severity of illness score; SIRS, systemic infammactery response syndrome score; SOFA, sequential organ failure assessment.


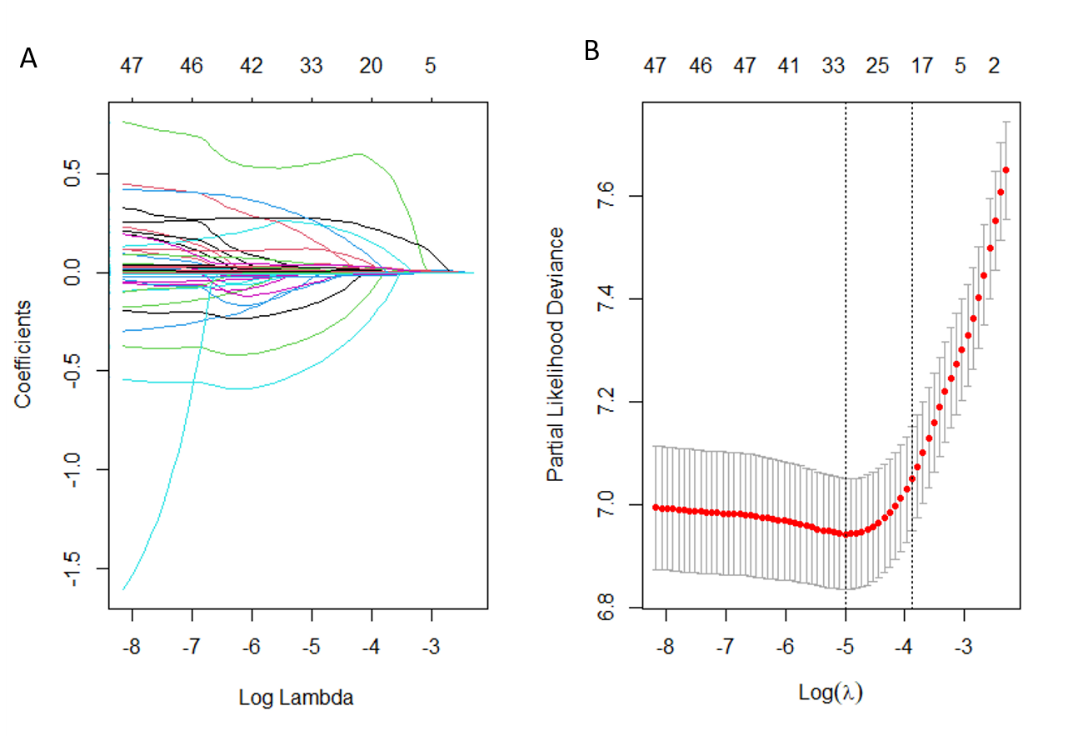


Figure. S1. LASSO analysis process. (A)LASSO coefficient profiles of variables. (B) Ten-fold cross-validation for tuning parameter selection in the LASSO analysis.

Notes: LASSO, the Least absolute shrinkage and selection operator.


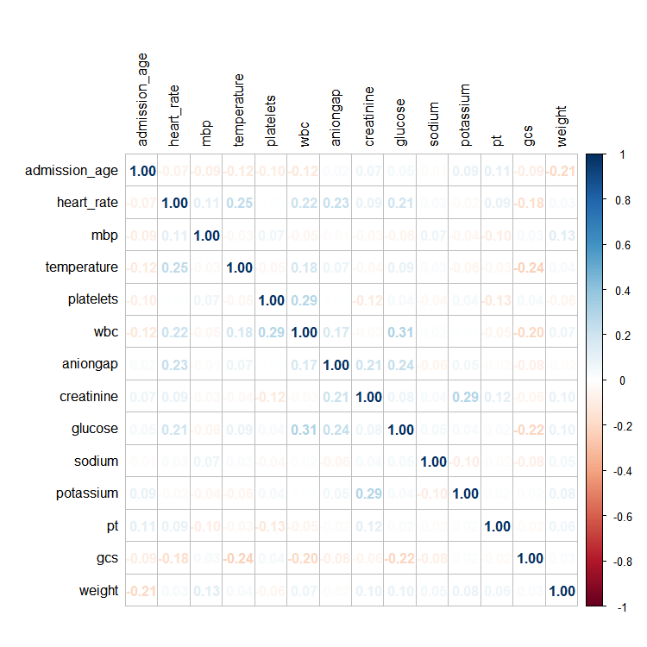


Figure. S2. Correlation matrix of selected continuous variables.

Notes: MBP, mean blood pressure; WBC, white blood cell; PT, prothrombin time; GCS, glasgow coma scale.


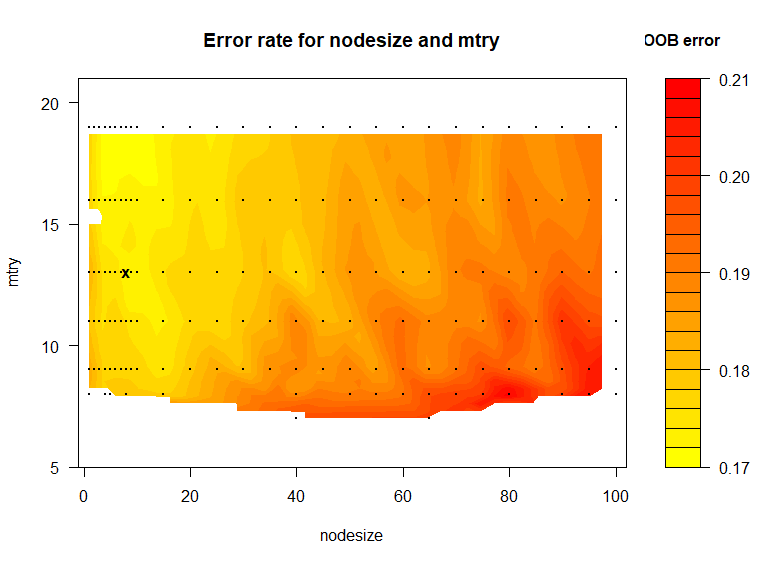


Figure. S3. Tuning parameters of RSF model by grid search method.


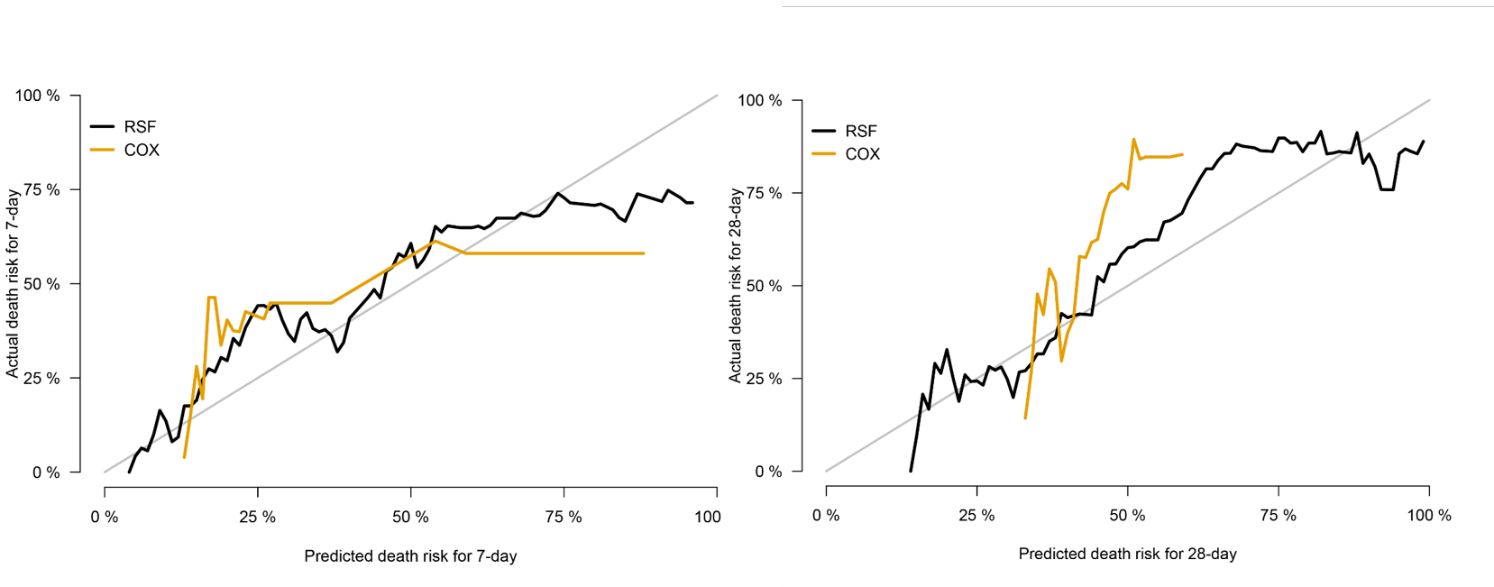


**Figure. S4.** Calibration curves of RSF and COX models for 7-day and 28-day.

Notes: RSF, Random survival forest.


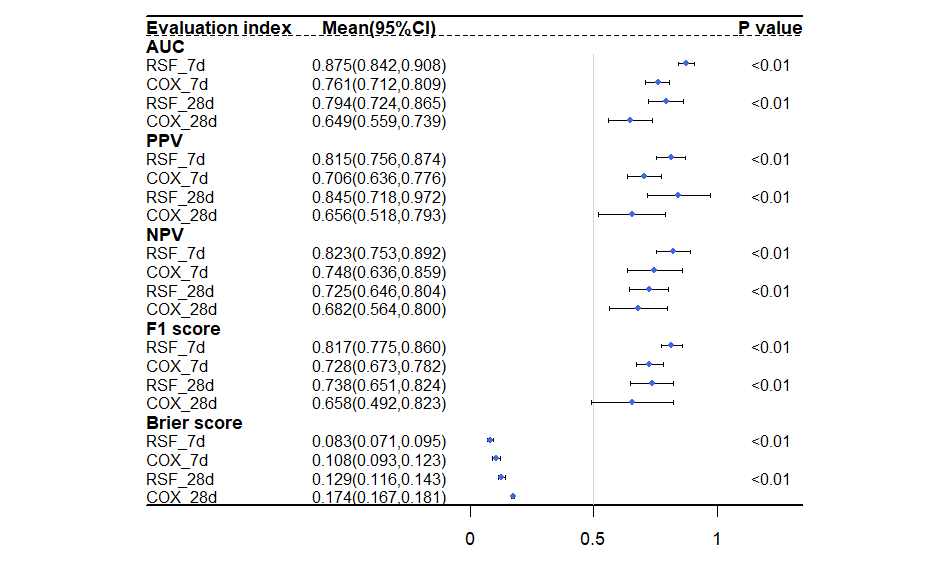


Figure. S5. Forest plot for model evaluation indicators.

Notes: RSF, Random survival forest; AUC, area under the curve; PPV, positive predictive value; NPV, negative predictive value.


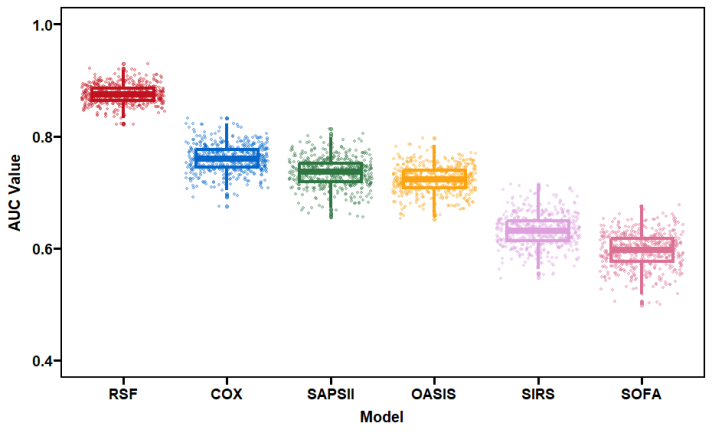


Figure. S6. Box scatter plot for comparison of RSF with traditional score systems.

Notes: AUC, area under the curve; RSF, Random survival forest; SAPSⅡ, simplified acute physiology score; OASIS, Oxford acute severity of illness score; SIRS, Systemic infammactery response syndrome score; SOFA, sequential organ failure assessment.

**Table S4** NPV and PPV of traditional scoring systems for 7-day mortality

| Models | PPV | NPV |
| --- | --- | --- |
| SAPSII | 0.702(95%CI 0.663-0.74) | 0.729(95%CI 0.661-0.798) |
| OASIS | 0.642(95%CI 0.593-0.691) | 0.790(95%CI 0.667-0.913) |
| SIRS | 0.608(95%CI 0.517-0.699) | 0.617(95%CI 0.531-0.703) |
| SOFA | 0.661(95%CI 0.575-0.746) | 0.590(95%CI 0.534-0.646) |

Notes: PPV, Positive predictive value; NPV, Negative predictive value; OASIS, Oxford acute severity of illness score; SOFA, sequential organ failure assessment; SAPSⅡ, simplified acute physiology score; SIRS, Systemic infammactery response syndrome score.

**Table S5.** The AUC values of six models for 28-day mortality.

| Models | AUC value |
| --- | --- |
| RSF | 0.794(95%CI 0.724-0.865) |
| COX | 0.649(95%CI 0.559-0.739) |
| SAPSII | 0.667(95%CI 0.577-0.757) |
| OASIS | 0.553(95%CI 0.463-0.643) |
| SIRS | 0.532(95%CI 0.443-0.621) |
| SOFA | 0.562(95%CI 0.478-0.646) |

Notes: AUC, area under the curve; RSF, Random survival forest; SAPSⅡ, simplified acute physiology score; OASIS, Oxford acute severity of illness score; SIRS, Systemic infammactery response syndrome score; SOFA, sequential organ failure assessment.
